# Report of Messieurs Cosnier, Maloet, Darcet, Philip, Le Preux, Desessartz, and Paulet, Doctors-Regent of the Faculty of Physic at Paris; Concerning the Acknowledged Advantages of the New Method of Administering Electricity in Nervous Disorders, Particularly in the Epilepsy and Catalepsy

**Published:** 1784

**Authors:** 


V. *Rapport de M. M. Cosnier, Maloet, Darcet, Philip, LePreux, Desessartz, et Paulet, docteurs-regens de la faculté de medecine de Paris; sur les avantages reconnus de la nouvelle méthode d'administrer l'electricité dans les maladies nerveuses, particulierement dans l'epilepsie, et dans la catalepsie; par M.* Ledru, *connú sous le nom de* Comus. *Lû a l'assemblée de cette faculté, dite du Prima Mensis, tenue au mois d'Avril dernier. Ce rapport est precedé de l'apperçu du systeme de l'auteur sur l'agent qu'il emploie, et des avantages qu'il en a tirés.* i. e. *Report of Messieurs Cosnier, Maloet, Darcet, Philip, Le Preux, Desessartz, and Paulet, doctors-regent of the faculty of physic at Paris; concerning the acknowledged advantages of the new method of administering electricity in nervous disorders, particularly in the epilepsy and catalepsy, by M.* Ledru, *known by the name of* Comus. *Read at a meeting of that faculty, called Prima Mensis, held in the month of April last. This report is preceded by a sketch of the author's theory of the agent he employs, and of the advantages he has derived from it.* 8vo. Paris, 1783. 115 pages. Printed at the expence of government.

THE

THE opinion at present most commonly entertained in this country, concerning medical electricity is, that the administration of strong shocks is, in general, not only useless but even hurtful; and Mr. Cavallo, one of the latest and most esteemed writers on this subject (*see our 1st vol.* p. 248.) contends, that the greatest electric powers which can be applied with success are exceedingly small shocks. But the cases related in the work before us are repugnant to this doctrine; as it appears, that the method of administering electricity in epileptic cases, adopted by the Sieur Ledru, consists in giving very strong shocks. In proof of the efficacy of this practice we have the testimony of the seven physicians whose names are mentioned in the title. The experiments were made on thirteen patients under the immediate inspection of these gentlemen in a house provided for this purpose by government. These cases were selected as the most inveterate of about five hundred epileptic and hysterical patients in the Bicetre and Salpetriere hospitals at Paris. Seven of the patients were males and the rest females. They are all said to have been cured in about eight or nine months; but neither the strength of the shocks nor the mode of administering them are afcertained with fufficient accuracy, as we are only told in general terms, that they were very ftrong.

The refult of thefe experiments are, 1. That electricity at firft renders the paroxyfms of epilepfy more frequent; thus the firft of the thirteen patients, a boy twelve years old, who had ufually had only about three fits every day, that is, 90 in 30 days, had 279 the firft month he was electrified.—2. That electricity after a certain time renders the fits lefs frequent: as a proof of this we may remark, that the patient juft now mentioned had only 63 fits the fecond month, and 14 in the third. The violence of the attack is likewife faid to be diminifhed when ftrong fhocks are adminiftered during the paroxyfm, as the fits, which when left to nature commonly lafted a quarter and even half an hour, were by thefe means fhortened to two, three, or at moft five minutes, and fometimes the fits, though preceded by the ufual fymptoms, were totally prevented.—3. That the paroxyfms are not only rendered lefs frequent, but alfo lefs violent.—4. That electricity, as adminiftered by Mr. Ledru, ftrengthens the mufcular fibre, and, notwithftanding the violence lence of the ſhocks, is productive of no inconvenience.

